# Genome-Wide Analysis Reveals Stress and Hormone Responsive Patterns of JAZ Family Genes in *Camellia Sinensis*

**DOI:** 10.3390/ijms21072433

**Published:** 2020-03-31

**Authors:** Jiazhi Shen, Zhongwei Zou, Hongqing Xing, Yu Duan, Xujun Zhu, Yuanchun Ma, Yuhua Wang, Wanping Fang

**Affiliations:** 1 College of Horticulture, Nanjing Agricultural University, Nanjing 210095, China; 2017204031@njau.edu.cn (J.S.); 2018804148@njau.edu.cn (H.X.); 2018204034@njau.edu.cn (Y.D.); zhuxujun@njau.edu.cn (X.Z.); myc@njau.edu.cn (Y.M.); wangyuhua@njau.edu.cn (Y.W.); 2 Department of Plant Science, University of Manitoba, Winnipeg, MB R3T 2N2, Canada; Zhongwei.Zou@umanitoba.ca

**Keywords:** *Camellia sinensis*, JAZ, abiotic stresses, expression profiles

## Abstract

JAZ (Jasmonate ZIM-domain) proteins play pervasive roles in plant development and defense reaction. However, limited information is known about the JAZ family in *Camellia sinensis*. In this study, 12 non-redundant *JAZ* genes were identified from the tea plant genome database. Phylogenetic analysis showed that the 12 JAZ proteins belong to three groups. The *cis*-elements in promoters of *CsJAZ* genes and CsJAZ proteins interaction networks were also analyzed. Quantitative RT–PCR analysis showed that 7 *CsJAZ* genes were preferentially expressed in roots. Furthermore, the *CsJAZ* expressions were differentially induced by cold, heat, polyethylene glycol (PEG), methyl jasmonate (MeJA), and gibberellin (GA) stimuli. The Pearson correlations analysis based on expression levels showed that the *CsJAZ* gene pairs were differentially expressed under different stresses, indicating that *CsJAZs* might exhibit synergistic effects in response to various stresses. Subcellular localization assay demonstrated that CsJAZ3, CsJAZ10, and CsJAZ11 fused proteins were localized in the cell nucleus. Additionally, the overexpression of *CsJAZ3*, *CsJAZ10,* and *CsJAZ11* in *E. coli* enhanced the growth of recombinant cells under abiotic stresses. In summary, this study will facilitate the understanding of the *CsJAZ* family in *Camellia sinensis* and provide new insights into the molecular mechanism of tea plant response to abiotic stresses and hormonal stimuli.

## 1. Introduction

Plants regulate their growth and developmental processes to adapt to various internal and external stimuli [1]. Phytohormones, a diverse group of signaling molecules found in small quantities in cells, including auxin (IAA), abscisic acid (ABA), ethylene (ET), gibberellins (GAs), salicylic acid (SA), methyl jasmonate (MeJA) and jasmonates (JAs) play critical roles in mediating these responses. Thus, phytohormones are considered as the most important endogenous substances for modulating physiological and molecular responses, which is essential for sessile plant growth and development [2]. Jasmonates, including JA and its bioactive derivatives, regulate many aspects of plant vital activities, such as growth, development, and defense [3,4]. Within the JA-triggered signaling transduction, the JAZ proteins play a centric role [5]. 

JAZ proteins are members of the plant-specific TIFY family that contained a conserved TIF[F/Y]XG sequence within the ZIM (also known as TIFY) motif near its N-terminal [6]. The other trait of JAZs is the highly conserved JAZ (also known as CCT_2) motif [7]. The JAZ motif serves as a protein–protein interaction surface, which is a requisite for the suppression of COI1 and MYC2 [4]. It has been reported that the JA signaling molecules, SCF^COI1^ complex, Jasmonate-ZIM (JAZ) domain repressor, and MYC2 transcription activator are involved in the JA signaling pathway and could interact with each other during the JA signaling process [8]. In response to stimuli, plant cells accumulate JA, which accelerates the combination of JAZ proteins and SCF^COI1^. The JAZ proteins are then ubiquitinated and alternatively degraded by 26S protease. Therefore, the JA response genes can transcript after the inhibition [9,10]. Thus, the COI1–JAZ–MYC2 model has been considered as the first central signal module in the JA pathway. In another model, the JAZ proteins recruit the repressors TPL (TOPLESS) and TPRs (TPL-related proteins) via an adaptor protein NINJA (novel interactor of JAZ) to repress the expressions of transcription factors (TFs) in the absence of the stimulation. Moreover, the JAZ proteins containing EAR motifs can recruit TPL independent of NINJA [11]. Additionally, JAZ proteins are also involved in other plant hormone biosynthesis or signaling pathways such as gibberellic acid, auxin, ethylene, and salicylic acid (SA) [12]. Thereby JAZ proteins are involved in the repression of multiple TFs and then inactivate their downstream responses, respectively [7,12,13].

Previously, 12, 15, 30, and 14 JAZ genes were found in *Arabidopsis thaliana* [14], rice [6], upland cotton [15], and wheat [10], respectively. In addition, the JAZs within a species have different biological functions. For example, JASMONATE-ASSOCIATED1 (*JAS1*), also known as *JAZ10*, could be induced by mechanical wounding via a COI1-dependent way in *Arabidopsis* [16]. Besides, *AtJAZ10* has been reported as a negative regulator not only in JA signaling but also in disease symptom development [17]. In addition, *AtJAZ2* was found to express only in stomata in which it triggered stomatal closure to prevent pathogen penetration [18]. *OsJAZ9* was acted as a transcriptional regulator in jasmonate signaling and could modulate salt stress or potassium deficiency tolerance in rice [19,20]. Otherwise, overexpression of *GhJAZ2* inhibited lint and fuzz fiber initiation and led to reduced fiber length [21]. *TaJAZ1* negatively regulated abscisic acid (ABA)-mediated seed germination inhibition and ABA-responsive gene expression and increased powdery mildew resistance in bread wheat [22,23]. Overexpression of *GsJAZ2*, a novel JAZ family gene from *Glycine soja*, could enhance *Arabidopsis* tolerance to salt and alkali stresses [24]. *NaJAZh* had been found to regulate a subset of defense response genes to resist herbivores and spontaneous leaf necrosis, while NaJAZd protein was essential for counteracting flower abscission in *Nicotiana attenuata* plants [25,26]. The *PnJAZ1* confers moss salinity tolerance through the abscisic acid signaling pathway [27]. Overexpression of *JAZ4* from wild grape in *Arabidopsis* improved resistance to powdery mildew through SA and/or JA signaling pathways [28]. In summary, JAZ genes presented important roles in regulating the adaptation or defense of biotic and abiotic stresses to maintain plant growth.

Tea plants (*Camellia sinensis* (L.) O. Kuntze) are crucial commercial perennial woody crops, which are widely cultivated in the world. Tea plants are grown in the tropic or subtropics areas [29,30]. Tea plants can easily be exposed to a variety of environmental stress factors such as extreme temperatures, high salt, and drought, which affect plant growth and development. Since jasmonate plays a critical role in modulating plant defenses, it is important to understand JA-mediated processes that contribute to tea plant stress tolerance. The released tea genome would allow us to conduct a genome-wide identification and analysis of the JAZ gene family in this woody species. In this study, we performed a bioinformatics analysis of the JAZ gene family in *Camellia sinensis*. Phylogenetic relations of the members of JAZ family, conserved domains and motifs, the transcriptional expression profiles of the *Camellia sinensis* JAZ genes in response to adversity stresses and hormones were analyzed. We also examined the subcellular localizations of three JAZ genes, *CsJAZ3*, *CsJAZ10,* and *CsJAZ11* in onion cells, and growth conditions of overexpression cells of these three genes under the abiotic stresses. This study lays a theoretical foundation for further exploration of the function of tea *CsJAZ* genes and may provide a new perspective for resistance breeding in tea plants.

## 2. Results

### 2.1. Genome-Wide Identification of JAZ Genes in Camellia Sinensis

To identify JAZ-related proteins in *Camellia sinensis*, the HMMER profiles of the TIFY domain (PF06200) and Jas domain (PF09425) was implemented against the genomes of the genome data of Shucahzao and Yunkang 10. After retrieving the database, a total of 12 full-length protein sequences were obtained. These genes were further analyzed using SMART and CD-search to ascertain the presence of TIFY and JAZ domains in the generated amino acid coding sequences. The length of encoded proteins ranged from 136 (CsJAZ6) to 436 (CsJAZ7) amino acid residues, and the molecular weight was distributed from 15.60 (CsJAZ6) to 46.21 (CsJAZ7) kDa. The isoelectric point (p*I*) of ten CsJAZ proteins was alkaline (p*I* > 7.0), while the other two proteins (CsJAZ1 and CsJAZ5) were acidic (p*I* < 7.0). The GRAVY scores indicated that all CsJAZ were hydrophilic proteins (GRAVY < 0; Table S1).

### 2.2. Phylogenetic Analysis and Sequence Alignment of CsJAZ

**Figure 1:**
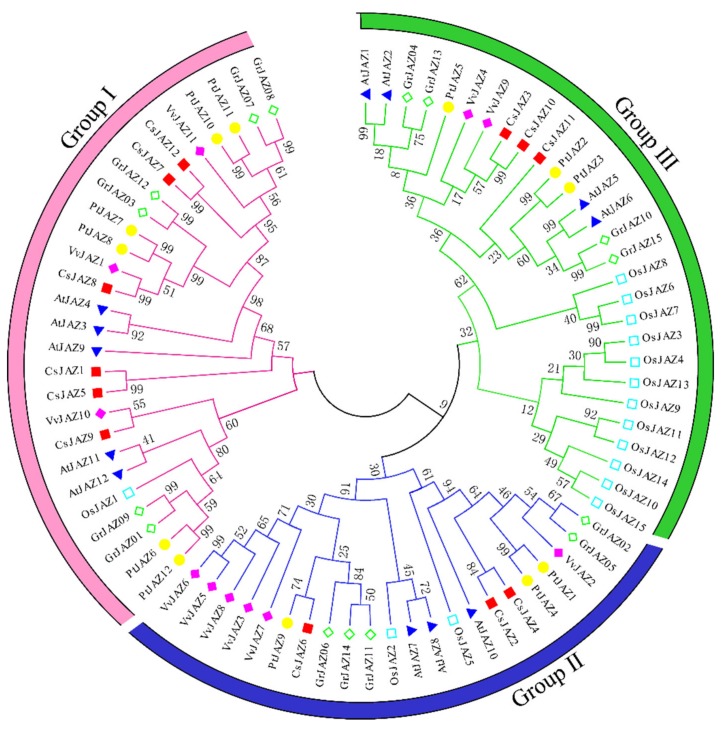
Phylogenetic relationship of JAZ proteins from *Camellia sinensis* (Cs), *Arabidopsis thiatina* (At), *Oryza sativa* (Os), *Vitis vinifera* (Vv), *Populus trichocarpa* (Pt), and *Gossypium raimondii* (Gr). The unrooted phylogenetic tree was constructed using MEGA 6 by the neighbor-joining method, and the bootstrap test was performed with 1000 iterations. The three groups are indicated with camber lines.

The full-length JAZs of *Arabidopsis thaliana*, *Oryza sativa*, *Vitis vinifera*, *Populus trichocarpa*, *Gossypium raimondii,* and *Camellia sinensis* were used to conduct a multiple sequence alignment, and a phylogenetic tree was constructed. The N-J tree was built using MEGA 7.0 software, and the reliability was tested by bootstrap analysis for 1000 replicates. As shown in Figure 1, all the JAZ proteins derived from different plants were clustered into three groups (Groups I–III). The twelve *Camellia sinensis* CsJAZs were clustered into three branches. Group I contained the maximal number of CsJAZ proteins, including CsJAZ1, CsJAZ5, CsJAZ7, CsJAZ8, CsJAZ9, and CsJAZ12. Group Ⅱ consisted of CsJAZ2, CsJAZ4, and CsJAZ6, while group III comprised CsJAZ3, CsJAZ10, and CsJAZ11. Interestingly, we found that the CsJAZ proteins clustered into the same clades with PtJAZ, VvtAZ, AtJAZ, or GrJAZ proteins rather than with OsJAZ proteins. The result revealed that the CsJAZ proteins shared high similarity with those in *Populus trichocarpa*, *Vitis vinifera*, *Arabidopsis thiatina*, *Gossypium raimondii* than those in *Oryza sativa*, which is consistent with the fact that the former five and tea plants are dicots and diverged more recently from a common ancestor than from the lineage leading to monocots.

The mode of amino acid residue conservation in all CsJAZ proteins were analyzed. The result showed that the 12 CsJAZs only represented 21.59% amino acid identity (data not shown), and the N-terminal and C-terminal regions of the CsJAZs were highly divergent (Figure S1). In the N-terminal of CsJAZ proteins, all CsJAZs members shared a conserved T(I/L)(F/S)(Y/F) sequence. Moreover, the multi-sequence alignments of identified JAZ proteins revealed that the amino acid sequence “TIFY” was changed into “TLSF” in CsJAZ1 and CsJAZ5. Besides the TIFY domain, the JAZ domains were also conserved, although there were different gene lengths and great sequence polymorphisms. As shown in Figure S1, α-helix regions of the JAZ domain were relatively conserved, but loop regions varied in all the tested JAZ genes. CsJAZ2 had a short conserved LPIARR motif, which has been reported to seal JA-Ile into its binding pocket at the COI1-JAZ interface. CsJAZ11 contained the canonical C-terminal end (IARR) of the motif that contact JA-Ile but lack the N-terminal (LP). Additionally, CsJAZ6 lacked the LPIARR motif and contained an EAR motif in the N-terminal.

### 2.3. Gene Structure and Conserved Motifs of CsJAZ Proteins 

To further examine the structural characteristic of the *CsJAZ* gene family, exon-intron distribution and conserved motifs were analyzed. The number of introns and exons in *CsJAZ* genes varied from 3 to 7, and 3 to 10, respectively (Figure S2). Particularly, five, three, two, and two genes had four, five, seven, and three introns, respectively. It is noteworthy that closely related genes in the phylogenetic tree had similar gene structural components, suggesting functional similarity within subgroups. 

A total of 16 conserved motifs (Figure S3A) in the 12 CsJAZ proteins were detected using the MEME web server. In general, JAZ proteins clustered in the same subgroups shared similar motif compositions, indicating functional similarities among members of the same subgroup. Motifs 1 and 2 contained JAZ (Figure S3B) and TIFY (Figure S3B) domains, respectively, and were found in all CsJAZ proteins. Motif 3 also appeared in all CsJAZ proteins. From Figure S3, we could clearly find that some motifs were present in different regions of the protein members in different groups. For example, Motif 5 was located on the right position of Motif 2 in CsJAZ1, CsJAZ3, CsJAZ5, and CsJAZ10. However, it was distributed on the left position of Motif 2 in CsJAZ2, CsJAZ4, CsJAZ7, CsJAZ8, and CsJAZ12. Motifs 4 and 6 were specifically distributed in the N-terminal regions of the corresponding proteins. Motifs 7, 8, 10, 11, 12, 13, 14, and 15 were found distributed in N-terminal or C-terminal regions in different group members. Motifs 9 and 16 were mainly distributed in the C-terminal regions of the corresponding proteins, except CsJAZ5 and CsJAZ9. The differences in motif distribution among the subgroups of JAZ genes revealed that the functions of these genes might have diverged during evolution.

### 2.4. Putative cis-Acting Regulatory Elements in the Promoter Region of CsJAZ *Genes*

**Figure 2:**
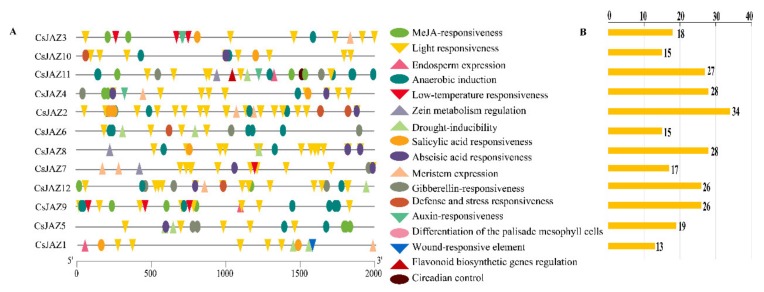
Predicted cis-elements that relate to abiotic stress in the *CsJAZ* promoters. (**A**) The distribution of cis-elements in the 2-kp upstream promoter regions of *CsJAZ* genes that related to abiotic stress responses are depicted. Different cis-elements are represented by different shapes and colors. (**B**) The number of abiotic stress response-related cis-elements in each *CsJAZ* gene promoter.

To obtain the information about the cis-acting regulatory elements of *CsJAZ* gene family, the putative promoter region sequence of each *CsJAZ* gene was analyzed. A total of 40 types of putative cis-acting regulatory elements were identified in *CsJAZ* genes, which were involved in 17 functions (Figure 2A). These regulatory elements included the TC-rich repeat element involved in defense and stress responsiveness, the LTR element involved in low-temperature responsiveness, MYB-binding sites (MBS) involved in drought-inducibility, the MYB binding site (MRE) and ACE involved in light responsiveness, the MYB binding site (MBSI) involved in flavonoid biosynthetic gene regulation, the ABRE associated with ABA signaling pathway, AuxRR-core and TGA elements related to Auxin-responsiveness, TCA-elements involved in salicylic acid-responsive elements, CGTCA and TGACG motifs involved in MeJA responsiveness, P-box, TATC-box, and GARE-motif involved in gibberellin-responsive. The type and number of cis-acting regulatory elements in the promoter region of each *CsJAZ* gene were different (Figure 2B, Table S2). For instance, the LTR element was only found in the promoter regions of *CsJAZ3*, *CsJAZ7,* and *CsJAZ9*. MBS was found in the promoter regions of *CsJAZ1*, *CsJAZ5*, *CsJAZ6*, *CsJAZ8*, *CsJAZ11,* and *CsJAZ12*. TGA-elements were presented in the promoter regions of *CsJAZ3* and *CsJAZ4*, while AuxRR-core was only presented in promoter regions of *CsJAZ11*. Noteworthy, MBSI was found in the promoter regions of *CsJAZ11* (Table S2). These showed that the different members of the *CsJAZ* gene family might be involved in different abiotic stresses.

### 2.5. Functional Analysis and Interaction Networks of CsJAZ Proteins

GO database revealed that the *JAZ* genes are involved in the two main categories (cellular component and biological process). GO terms from “cellular component” contained “nucleus”. GO terms from “biological process” were involved with five processes response to stimuli, including defense response (GO0006952), jasmonic acid-mediated signaling pathway (GO0009867), regulation of defense response (GO0031347), regulation of signal transduction (GO0009966), and response to wounding (GO0009611) (Table S3). In addition, all CsJAZ proteins were investigated in an *Arabidopsis* association model in STRING software to identify the functional interactions (Figure S4). Protein interaction analysis indicated that twelve CsJAZ proteins interacted with each other. They could also interact with other genes with regulation function, such as MYC2, MYC3, and COI1 (Figure S4). 

### 2.6. Expression Analysis of CsJAZ Genes in Different Tissues

**Figure 3:**
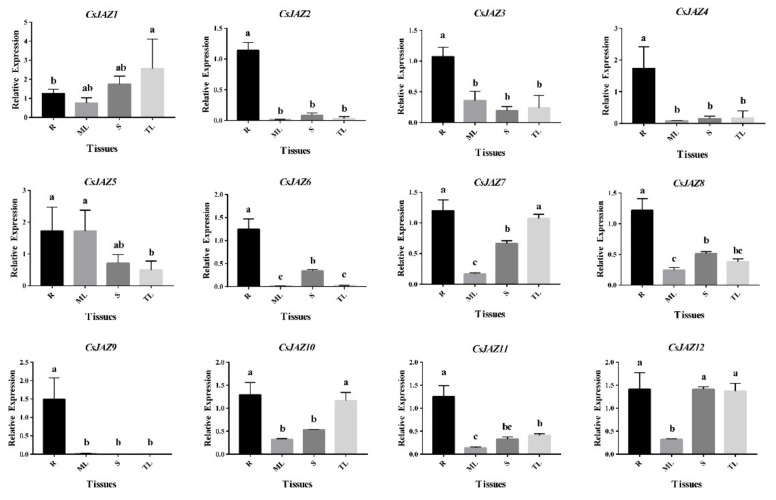
Quantitative RT–PCR analysis of expression of *CsJAZ* genes in different tea tissues. Data presented in the quantitative RT–PCR analysis were mean values and standard deviation of three biological replicates of different tissues and three technical replicates in each biological sample. The *y*-axis is the relative expression level. The different lower case letters above bars indicated the signifcance (*p* < 0.05) of the relative expression level between two samples. R: roots; ML: mature leaves; S: stems; TL: tender leaves.

Generally, all the *CsJAZ* genes were expressed in roots, mature leaves, stems, and tender leaves (Figure 3). Most *CsJAZ* genes showed higher expression in roots than that in leaves and stems. Moreover, six genes (*CsJAZ2*, *CsJAZ3*, *CsJAZ4*, *CsJAZ6*, *CsJAZ8*, *CsJAZ9,* and *CsJAZ11*) were highly expressed in the root and four genes (*CsJAZ5*, *CsJAZ7*, *CsJAZ10,* and *CsJAZ12*) were highly expressed in both the root and one or two of the other three tissues, while *CsJAZ1* was highly expressed in tender leaves and other tissues. Interestingly, further analysis showed a relative relationship between the tissue expression and evolution of *CsJAZ* genes. For example, *CsJAZ2*, *CsJAZ4,* and *CsJAZ6* in Group Ⅱ showed similar gene expression patterns, indicating that these gene expression levels were higher in stem than in leaves. *CsJAZ7* and *CsJAZ12* in Group I showed a similar gene expression patterns, indicating that these gene expression levels were lowest in mature leaves. Interestingly, genes *CsJAZ1*, *CsJAZ5,* and *CsJAZ9* in Group I had close phylogenetic relationships and inconsistent tissue expression patterns with each homologous gene pair. These results suggest that *CsJAZ* genes have different expression patterns and tissue specificity.

### 2.7. Expression Analysis of CsJAZ Genes under Abiotic Stresses

**Figure 4:**
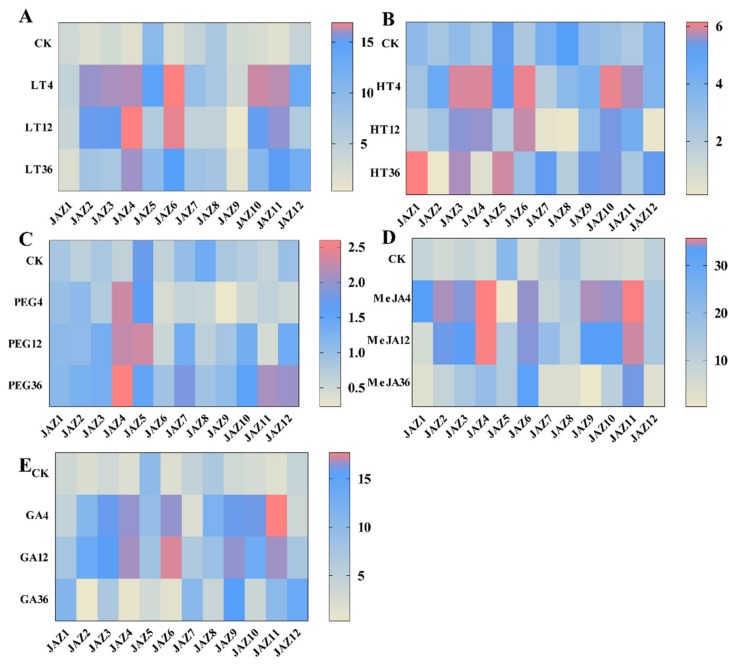
Expression heat maps of 12 *CsJAZ* genes under 5 treatments (low temperature (LT), high temperature (HT), polyethylene glycol 6000 (PEG 6000), jasmonate (MeJA), and gibberellin (GA)) in tea plants “Longjing43”. qRT–PCR strategy was used to analyze the relative expression level of each *CsJAZ* gene. A Expression heat maps of 12 *CsJAZ* genes under LT stress. B Expression heat maps of 12 *CsJAZ* genes under HT stress. C Expression heat maps of 12 *CsJAZ* genes under 10%PEG 6000 stress. D Expression heat maps of 12 *CsJAZ* genes under 100 µM MeJA treatment. E Expression heat maps of 12 *CsJAZ* genes under 100 µM GA treatment. The expression level of tea actin was used as the internal control to standardize the RNA samples for each reaction and the expression at 0 h was set as CK. The labels on the left side of each heatmap indicated the samples with different treatments and sampling time after treatments. Relative expression levels were shown in color as the scale. The data were from three biological replicates.

In this study, the expression patterns of the *CsJAZ* genes were detected by qRT–PCR, and the results revealed obvious differences under low temperature, high temperature, water deficit (10% PEG 6000), and MeJA and GA treatments with different exposure time. We noticed that the *CsJAZ* genes were regulated at the transcriptional level by the low temperature (4 °C; Figure 4A). For example, the transcriptional levels of *CsJAZ2*, −*3*, −*4*, −*6*, −*10,* and −*11* were highly upregulated at each time point of the cold stress and exhibited several fold upregulation compared to the control. Except for *CsJAZ4,* these upregulated *CsJAZ* genes showed the highest transcript levels at 4 h and exhibited a steady decrease expression from 4 to 36 h. *CsJAZ4* showed the highest transcript level at 12 h. *CsJAZ5*, −*7*, −*8,* and −*12* genes showed a steady decrease expression from 4 to 12 h and then an increasing expression from 12 to 36 h. These results suggest that the highly upregulated JAZ genes in tea plants might be related to cold tolerance.

Under high-temperature stress (42 °C), most of the *CsJAZ* genes were acutely down- or upregulated in response to heat stress (Figure 4B). Four genes, including *CsJAZ3*, −*6*, −*10,* and −*11* genes were highly upregulated at each time point. There also existed four genes, *CsJAZ1*, −*5*, −*7,* and −*12*, the expression patterns of which showing an initial decrease then increase tendency throughout the high-temperature treatment period. These four genes presented the lowest transcript levels at 12 h and showed the highest levels at 36 h. *CsJAZ8* also showed an initial decrease, then subsequent increase tendency throughout the high-temperature treatment period, although the level of the upregulation was only about half as high as the control at 36 h. *CsJAZ2* and *4* showed the highest transcript levels at 4 h and exhibited a steady decrease from 4 to 36 h, and the expression levels were lower than the control. 

Under the water deficiency, which was simulated by PEG treatment, we observed that most of the *CsJAZ* genes exhibited an initial decrease then increase tendency throughout the PEG treatment period (Figure 4C). These genes were *CsJAZ3*, −*6*, *−7*, −*8*, −*9*, −*10,* and −*12.* Except for *CsJAZ8*, the other six genes showed the highest levels at 36 h. However, the expression level of *CsJAZ8* was lower than the control at 36 h. For *CsJAZ5*, the highest expression level was at 12 h. *CsJAZ1* and −*2* were upregulated to lower levels in response to water deficit stress during the PEG treatment period. For *CsJAZ4* and −*11*, the expression levels increased from 0 to 4 h and then decreased from 4 to 12 h. After that, their expressions increased from 12 to 36 h again, and the levels were higher than the control at 36 h. 

Following treatment with MeJA, JAZ genes in tea plants showed variable transcript abundance at different time points (Figure 4D). Most JAZ genes were rapidly upregulated and subsequently downregulated after receiving exogenous MeJA signals. Among the JAZ genes in tea plants, *CsJAZ4* and −*11* displayed the most prominent changes in expression levels. The expression levels of *JAZ4* and −*11* were upregulated more than 50-fold within 12 h of MeJA treatment and subsequently returned to a relatively lower level at 36 h after MeJA treatment. Strains of JAZ genes, such as *CsJAZ2*, −*3*, −*6*, −*9* and −*10* showed the same response pattern as *CsJAZ4* and −*11*, but their upregulation degrees were smaller than that of *CsJAZ4* and −*11.* In contrast, four JAZ genes, including *CsJAZ1*, −*5*, −*7,* and −*12* displayed a low degree of expression fluctuation and variable expression after MeJA treatment. However, the expression levels of *CsJAZ8* gene was downregulated after MeJA treatment. 

In response to GA treatment, most of the JAZ genes, including *CsJAZ4*, −*6*, −*8*, −*9*, −*10,* and −*11*, were induced at 5 and 12 h, with the highest upregulation shown for *CsJAZ11* (>17-fold) at 5 h (Figure 4E). In addition, these genes subsequently decreased over 12 to 36 h. Conversely, *CsJAZ5* was downregulated completely after exogenous GA treatment. The remaining genes were upregulated at least one time point in response to GA. These revealed that all CsJAZ genes could respond to various plant hormones, and they might be involved in some complex signaling pathways.

### 2.8. Correlations and Coregulatory Networks of CsJAZ Genes

**Figure 5:**
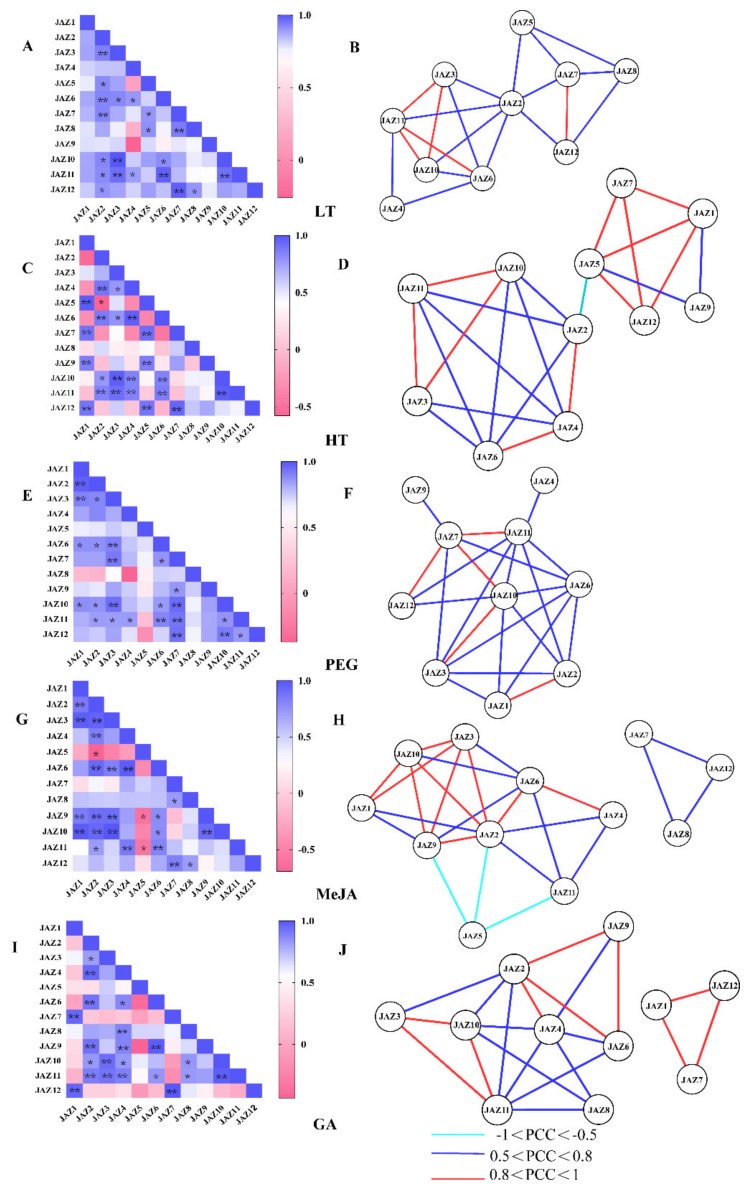
Correlations among *CsJAZ* genes under LT, HT, PEG, MeJA, and GA treatments. Correlation analysis of *CsJAZ* genes under LT (**A**), HT (**C**), PEG (**E**), MeJA (**G**), and GA (**I**) treatment was performed based on the PCCs of gene pairs calculated using GraphPad Prism 7 software. Correlations are indicated by the color scales. The lower bar represents the PCC values. * and ** represent correlations with *p*-value ≤0.05 and *p*-value ≤0.01, respectively. The co-regulatory network of *CsJAZ* under LT (**B**), HT (**D**), PEG (**F**), MeJA (**H**), and GA (**J**) treatment was illustrated by Cytoscape. The significant PCCs of gene pairs (*p*-value ≤0.05) are included, and the different correlation levels of the gene pairs are marked by edge lines with different colors, as shown below the coregulatory networks.

Co-expression network analysis is one of the powerful measures for predicting gene functions and functional modules [31]. To investigate the connections among these genes in response to LT, HT, PEG, MeJA and GA, correlation and coregulatory networks were established based on the PCCs of their relative expression levels. Significantly positive correlations between *CsJAZ* genes was for LT, HT, PEG, MeJA and GA treatments (Figure 5A,C,E,G,I). For example, positive correlations existed among *CsJAZ2*, -*3*, *-4*, *-5*, *-6*, *-7*, -*8*, *-10*, *-11,* and *-12* under LT treatment (Figure 5A). However, no significantly negative correlation was observed for *CsJAZ* genes under LT treatment (Figure 5A). The expression of *CsJAZ2* was significantly negatively correlated with *CsJAZ5* under HT (Figure 5C). There was no significantly negative correlation for *CsJAZ* genes under PEG treatment (Figure 5E). *CsJAZ2* was significantly negatively correlated with *CsJAZ5* under MeJA treatment (Figure 5G). Besides, *CsJAZ5* was also significantly negatively correlated with *CsJAZ9* and *CsJAZ11* under MeJA treatment (Figure 5G). 

Subsequently, a LT-related co-regulatory network of *CsJAZ* genes was constructed with 20 edges and 10 nodes (Figure 5B). All these gene pairs showed positive correlations (*p*-value ≤0.05, and 0.5 < PCC < 1.0). Among these positive correlations, 5 gene pairs showed stronger positive correlations (*p*-value ≤ 0.05, and 0.8 < PCC < 1.0), including *CsJAZ3* and *CsJAZ10*, *CsJAZ3* and *CsJAZ11*, *CsJAZ6* and *CsJAZ11*, *CsJAZ7* and *CsJAZ12*, *CsJAZ10* and *CsJAZ11*. 

There were 11 nodes with 24 edges in the HT-related co-regulatory network (Figure 5D). Except *CsJAZ2* and *CsJAZ5* gene pairs, the other gene pairs showed positive correlations (*p*-value ≤ 0.05, and 0.5 < PCC < 1.0). Among the positive correlations, *CsJAZ1* and *CsJAZ5*, *CsJAZ1* and *CsJAZ7*, *CsJAZ1* and *CsJAZ12*, *CsJAZ2* and *CsJAZ4*, *CsJAZ3* and *CsJAZ10*, *CsJAZ3* and *CsJAZ11*, *CsJAZ4* and *CsJAZ6*, *CsJAZ5* and *CsJAZ7*, *CsJAZ5* and *CsJAZ12*, *CsJAZ7* and *CsJAZ12*, *CsJAZ10* and *CsJAZ11* presented stronger positive correlations (*p*-value ≤ 0.05, and 0.8 < PCC < 1.0).

Ten nodes with 23 edges were in the PEG-related co-regulatory network (Figure 5F), and similarly, all gene pairs showed positive correlations in the ABA-related co-regulatory networks (*p*-value ≤0.05 and 0.5 ≤ PCC < 1). *CsJAZ1* and *CsJAZ2*, *CsJAZ3* and *CsJAZ10*, *CsJAZ7* and *CsJAZ10*, *CsJAZ7* and *CsJAZ11*, *CsJAZ7* and *CsJAZ12* presented stronger positive correlations (*p*-value ≤0.05 and 0.8 < PCC < 1.0).

There were 12 nodes with 25 edges in the MeJA-related co-regulatory network and 11 nodes with 21 edges in the GA-related co-regulatory network. Both networks contained two modules (Figure 5H,J). *CsJAZ2* and *CsJAZ6*, *CsJAZ3* and *CsJAZ10*, *CsJAZ3* and *CsJAZ11*, *CsJAZ10* and *CsJAZ11* also exhibited stronger positive correlations in the two coregulatory networks. In addition, the *CsJAZ2–CsJAZ5*, *CsJAZ5–CsJAZ9* and *CsJAZ5–CsJAZ11*, pairs exhibited significant negative correlations under MeJA treatment (*p*-value ≤0.05 and PCC < -0.5). 

### 2.9. Cloning of CsJAZ3, -10, and -11 and Subcellular Location

**Figure 6:**
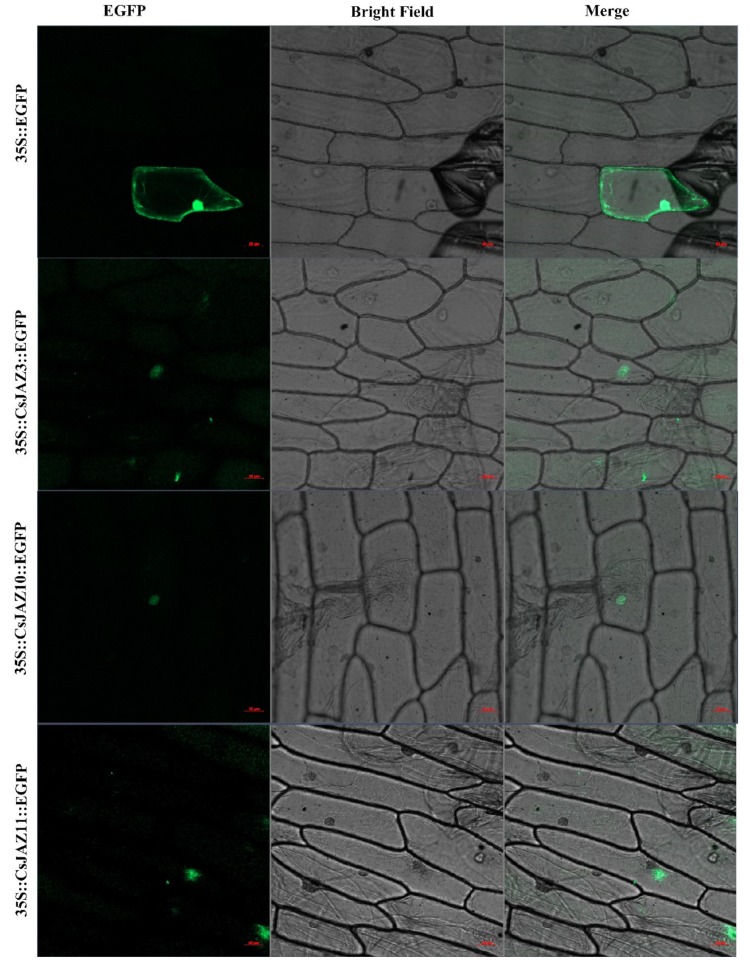
Subcellular localization of the fusion protein 35S::CsJAZ::EGFP in onion pidermal cells. The vector 35S::EGFP was used as the control. Bar = 50 μm. The green indicated where the proteins were located. The red line indicated the bar was 50 μm.

To investigate the reliability of identified *CsJAZ* genes, we used the specific primers to clone the ORF sequences of *CsJAZ3*, -*10,* and -*11* and confirmed these sequences by DNA sequencing. The results showed that the ORF length and the nucleic acid constituent of *CsJAZ3*, -*10,* and -*11* were in line with the data that deposited in the tea genome database (Figure S5). Subcellular localization indicated that CsJAZ3–EGFP, CsJAZ3–EGFP and CsJAZ11–EGFP fusion proteins were gathered in the nucleus (Figure 6), which is consistence with the prediction from CELLO. The finding indicated that these three JAZ proteins are nuclear localization proteins.

### 2.10. Overexpression of CsJAZ3, -10, and -11 in E. coli Enhanced its Growth under Abiotic Stresses

**Figure 7:**
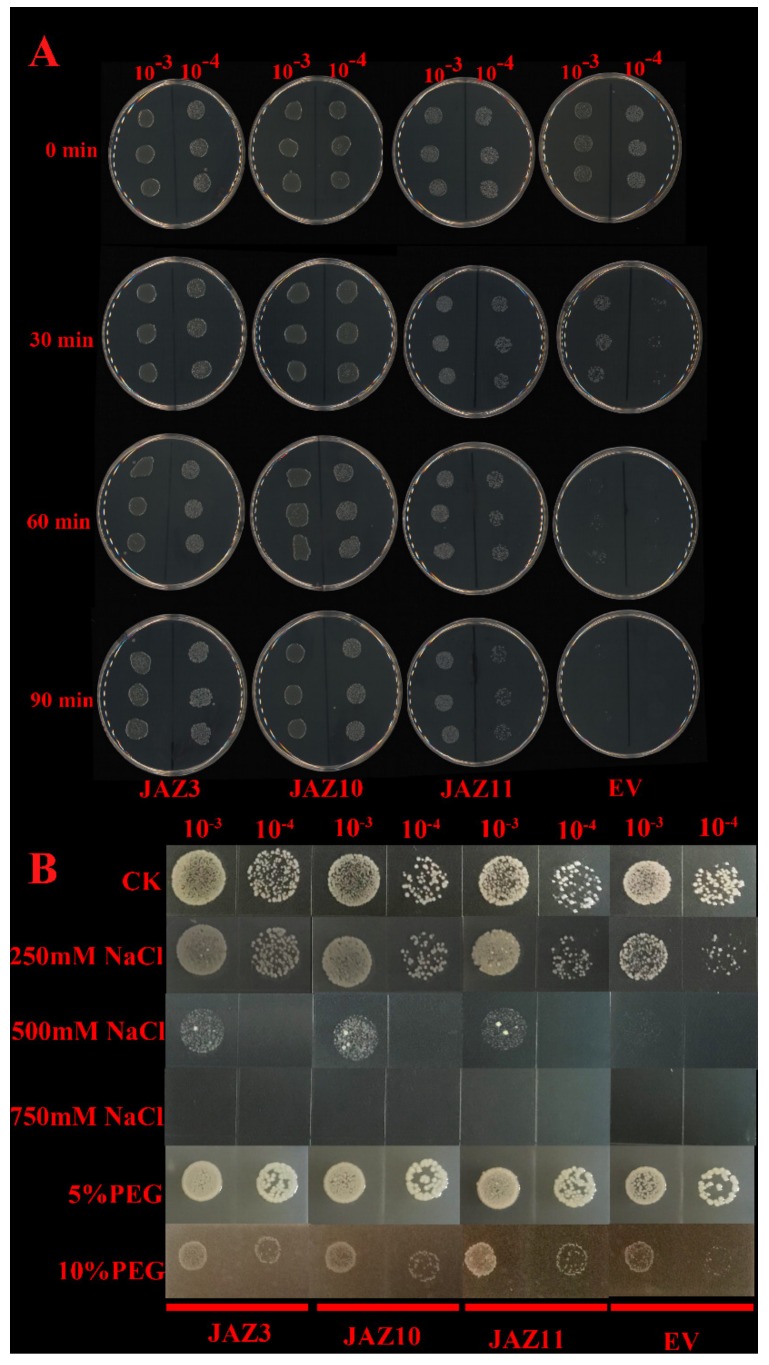
(**A**) Spot assay of BL21(DE)/pGEX-4T-1–CsJAZ3, pGEX-4T-1–CsJAZ10, and pGEX-4T-1–CsJAZ11 and BL21(DE)/pGEX-4T-1 (EV) on LB plates after the cell mediums were treated with 55 °C for 0, 30, 60, and 90 min. (**B**) Spot assay of BL21(DE)/pGEX-4T-1–CsJAZ3, pGEX-4T-1–CsJAZ10, and pGEX-4T-1–CsJAZ11, and BL21(DE)/pGEX-4T-1 (EV) on LB plates with NaCl and PEG.

There was no obvious difference in the growth rates of the pGEX-4T-1 and pGEX-4T-1–*CsJAZ3*, pGEX-4T-1–*CsJAZ10*, and pGEX-4T-1–*CsJAZ11* strains before heat treatments (0 min), indicating that *CsJAZ3*, *CsJAZ10* and *CsJAZ*11 overexpression did not affect *E. coli* growth under normal conditions (Figure 7A). Under heat stresses, the pGEX-4T-1 *E. coli* survival decreased rapidly, but the *CsJAZ3*, *CsJAZ10,* and *CsJAZ*11-overexpressing cells were more viable than the control cells. After a 90-min heat treatment, the pGEX-4T-1 cells almost all died, whereas pGEX-4T-1–*CsJAZ3*, pGEX-4T-1–*CsJAZ10*, and pGEX-4T-1–*CsJAZ11* strains cells showed better growth (Figure 7A). This observation suggests that *CsJAZ3*, *CsJAZ10,* and *CsJAZ*11 increased the thermotolerance of the transgenic *E. coli* cells. 

The *CsJAZ3*, *CsJAZ10,* and *CsJAZ*11-expressed cells were able to endure high salt concentrations of up to 500 mM NaCl. In contrast, the growth of the control cells was inhibited at 250 mM NaCl, and at 500 mM NaCl was proved lethal to the control (Figure 7B). After 24 h of cultivation, the BL21 *CsJAZ3*, *CsJAZ10,* and *CsJAZ*11-expressed cells showed a faster growth rate than that of the control, which was supplemented with PEG6000 (Figure 7B). The result showed that recombinant proteins enhanced cell growth under PEG stress.

## 3. Discussion

Jasmonate ZIM-domain (JAZ) proteins are central in the signal transduction cascade triggered by (+)-7-iso-jasmonoyl-L-isoleucine (JA-Ile) [12]. They act as transcriptional repressors of JA-responsive genes. JAZ family has been widely reported to play an important role in the growth and developmental processes in various plant species. However, research on the JAZ gene family has not been fully explored in tea plants. In this study, a comprehensive genome-wide analysis of the *CsJAZ* gene family in tea plant was carried out, and the results will provide a powerful theoretical foundation for future functional studies. 

Twelve CsJAZ proteins with highly conserved JAZ and TIFY domains were identified in *C*. *sinensis*. The number and length of exon and intron showed great variations in *CsJAZ* genes. This finding was consistent with the *JAZ* genes of other plants, such as upland cotton and wheat [10,15]. The structure variations of *JAZ* genes might be caused by structural divergence mechanisms, such as the insertion or deletion of the exons and introns proposed by Xu et al. [32]. Interestingly, most of the CsJAZs were basic proteins with p*I* values greater than 7, except for CsJAZ1 and CsJAZ5, which were with a pI value of 4.57 and 5, respectively. This may be because of CsJAZ1 and CsJAZ5 containing a higher proportion of acidic amino acids. In detail, 27 aspartic acids and 25 glutamic acids were in CsJAZ1, and 13 aspartic acids and 19 glutamic acids were in CsJAZ5, which were more than those present in the other CsJAZ proteins. The principle of the prediction of protein potential function depended on structural similarities. Based on homology alignments, CsJAZ members with similar motifs were classified into a group. Previous studies had reported that some *Arabidopsis* JAZ proteins were intertwined in protein–protein interactions and formed dimers interacting with other types of proteins [33]. For example, MYB21 and MYB24, two R2R3- transcription factors, were found to interact with JAZ1, JAZ8, and JAZ11 in both yeast and *Arabidopsis* [34]. Besides, the JAZ1 proteins were reported to interact with the WD-Repeat/bHLH/MYB complexes and then regulate jasmonate-mediated anthocyanin accumulation and trichome initiation in *Arabidopsis* [35]. According to the homology alignments analysis, CsJAZ3, -10, -11 were AtJAZ1-like protein that could interact with numerous JAZs in a predicted interactional network. The promoter analysis showed that the promoter of JAZ11 contained a flavonoid biosynthetic genes regulation element. Therefore, future functional verification of CsJAZ3, -10, -11 is needed to prove the functional interaction in flavonoid or anthocyanin accumulation. 

Expression profile analyses revealed that 12 *CsJAZ* genes expressed in all the examined tissues. It had been reported that the *JAZ* genes could be widely expressed in the plant tissues. In S*alvia miltiorrhiza*, constitutive expression of *SmJAZ1* and *SmJAZ2* was detected in roots, stems, and leaves [36]. In cotton, the JAZ genes widely expressed in various tissues, including roots, leaves, petals, anthers, and early stages of developing ovules and fibers [31]. In wheat, five *TaJAZ* genes (*TaJAZ1*, *-4*, -*10*, -*11,* and -*14*) were expressed in root, stem, leave, stamen, and pistil tissues [10]. To our surprise, most *CsJAZ* genes showed higher expression in roots than in leaves and stems. JAZ genes also showed high expression in the roots in several other plant species. For example, the *PtJAZ7*, *PtJAZ8,* and *PtJAZ12* in polar were found highly expressed in the roots [37]. In wheat, *TaJAZ6* and *13* were specifically expressed in root, and the expression level of *TaJAZ3* in root tissues was as high as that in stamen [10]. *GhJAZ1-A/D*, *GhJAZ3-A/D*, *GhJAZ5-A/D,* and *GhJAZ13-A/D* in the upland cotton also showed higher expression in roots than in stems or leaves [15]. These results indicated that *CsJAZ* genes might exert certain functions as *SmJAZ1* and *SmJAZ2* [36] in roots and maintain the normal development of tea plants under normal conditions. Taken together, the tissue-specific expression of *CsJAZ* indicated the possible roles of different group members in the various stress reaction and developmental processes.

Promoters located at the upstream of genes and play key roles in regulating the gene expression under stresses, for promoter regions contain a number of stress-related *cis*-elements that can combine with transcription factors [38]. To gain more insights into the function of *CsJAZ* genes, we analyzed the *cis*-acting regulatory elements composition and the expression patterns under various treatments. A number of stress-related and regulatory elements that can respond to plant hormones (IAA, ABA, MeJA, SA, and GA) abundantly enriched in *CsJAZ* promoters. These *JAZ* gene promoter regions were mainly different in the number and type of *cis*-acting regulatory elements. For example, the promoter region of *CsJAZ11* contained three CGTCA-motif, one MBSI, and one MBS, while *CsJAZ3* contained two CGTCA-motif and MBSI or MBS was an absence in it. 

The promoter sequences of certain *CsJAZ* genes lacked some types of *cis*-acting regulatory elements, but the expression patterns revealed that all *CsJAZ* genes could respond to all five treatments. For example, we could not find high temperature response-related *cis*-acting regulatory elements in each *CsJAZ* gene of tea plants. However, the relative expression levels of all *CsJAZ* genes were up- or downregulated upon high-temperature stress. Similarly, the promoter region of *TaJAZ* genes contained no high salinity response-related *cis*-acting regulatory elements, but all the *TaJAZ* genes could respond to the high salinity treatment [10]. It was speculated that the *CsJAZ* genes induced by a specific stressor do not have corresponding *cis*-elements in their promoters. This might indicate that the gene expression level under different treatments was not only determined by the presence of relevant *cis-*acting regulatory elements in the promoter region, but also by other physiological pathways in tea plants.

The TIFY family genes have been proven to be important TFs with various stress resistance [17]. Specifically, the JAZ subfamily genes were the best described responsive members to stresses to date [39]. For example, rice *JAZ* genes were reported to be induced by abiotic stresses such as low temperature, drought, and salinity, and one of them *OsTIFY11a* was found to enhance stress tolerance [6]. Likewise, *JAZ* genes were found to show different upregulation responses to cold, salt, and drought stresses in *B. rapa* [40]. Like the *JAZ* genes responding to the stresses in other plants, almost all the *CsJAZ* genes identified in this study showed upregulation to cope with the cold, heat, or osmotic stresses. There were six ideal candidates of *CsJAZ* genes (*CsJAZ2*, *-3*, *-4*, *-6*, *-10*, and *-11*) to be involved in a rapid and intense response to low temperatures. Except for *CsJAZ2*, the other five, along with *CsJAZ1* and *CsJAZ5,* also responded quickly and intensively to heat stress. *CsJAZ4*, *-5*, *-7*, -*10*, *-11* and *-12* showed a severe response to osmotic stress. Of the three treatments, cold stress induced relatively more fluctuations in the transcript abundance of *CsJAZ* genes than that induced by heat and PEG stresses. This result was different, with the findings in cotton that heat and salinity stresses induced relatively more fluctuations in the transcript abundance of *GhJAZ* genes than the cold and PEG stresses did [31]. In addition, the overexpression of *CsJAZ3*, *CsJAZ10,* and *CsJAZ11* in *E. coli* enhanced recombinant cell growth under abiotic stresses including high temperature, NaCl, and PEG, which indicated that *CsJAZ* genes played important roles in resistance to abiotic stresses. To investigate the connections among *CsJAZ* genes under different stresses, co-regulatory networks were established based on the PCCs of the relative expression levels of the genes. A series of *CsJAZ* gene pairs showed significant expression change correlations in the LT-related, HT-related, and PEG-related co-regulatory networks. The results showed common positive correlations of *CsJAZ* genes under different treatments, such as correlations between *CsJAZ3* and *CsJAZ10*, and *CsJAZ7* and *CsJAZ12* under LT, HT, and PEG treatments, and correlations among *CsJAZ3*, *CsJAZ10,* and *CsJAZ11* under LT and HT stresses. Negative correlations between *CsJAZ* gene pairs were also found, such as *CsJAZ2* and *CsJAZ5* in the HT-related coregulatory network. Thus, the correlations among *CsJAZ* genes varied under different conditions, indicating that *CsJAZ* genes might exhibit synergistic effects in response to various stresses. 

In responding to different phytohormones, *JAZ* genes seemed to have comprehensive regulatory impacts in various processes of plant development than those acted by other TIFY subfamily members [12]. JA treatment and environmental factors rapidly trigger JAZ gene expression, which might be responsible for moderating the response to JA [39]. Moreover, JAZ proteins also mediate JA-gibberellin (GA) and JA-ethylene (ET) synergistic crosstalk by interacting with DELLA proteins [41] or with the EIN3 and EIL1 transcription factors [42], respectively. For instance, *VvJAZ4*, *VvJAZ5*, and *VvJAZ9* in *Vitis vinifera* were upregulated by both JA and MeJA [43]. *ScJAZ1*–*ScJAZ7* genes in sugarcane were also induced by MeJA [44]. Likewise, the relative expression levels of most of the *TaJAZ* genes in *Triticum aestivum* were upregulated after GA treatment [10]. In our study, we found that in response to hormone treatments, most of the tea plant *JAZ* genes were highly upregulated after JA and GA treatments for 4 or 12 h. However, not each of these upregulated genes contained the corresponding *cis*-elements in their promoters like CGTCA-motif, TGACG-motif, GARE-motif, P-box, or TATC-box. For example, *CsJAZ2* and *CsJAZ6* did not contain *cis*-acting regulatory elements involved in the MeJA-responsiveness. However, they showed strong upregulation after MeJA treatment for 4 or 12 h. Similarly, *CsJAZ3*, *CsJAZ9,* and *CsJAZ10* lacked a Gibberellin-responsive element, whereas they showed strong upregulation after MeJA treatment for 4 or 12 h. Therefore, we assumed that the five genes responded by other unknown regulative pathways to MeJA or GA treatments. Additionally, the coregulatory networks showed that a positive correlation between *CsJAZ3* and *CsJAZ10* occurred in both MeJA-related and GA-related co-regulatory networks. Nevertheless, *CsJAZ11* presented no direct correlations with *CsJAZ3* or *CsJAZ10* in the MeJA-related co-regulatory network, whereas it presented significant positive correlations with *CsJAZ3* or *CsJAZ10* in the GA-related coregulatory network. It indicated that the *CsJAZ* genes exert similar or different functions responding to hormonal signals. In summary, we speculate that certain *CsJAZ* genes could respond to several different phytohormones, and they might play critical roles directly or indirectly in complex signaling pathways.

## 4. Materials and Methods 

### 4.1. Identification of JAZ Genes in Camellia Sinensis

To identify *JAZ* genes in tea plants, the whole-genome data of *Camellia sinensis* var. assamica Yunkang 10 and var. cultivar Shuchazao were downloaded from the website http://www.plantkingdomgdb.com/tea_tree/ and http://tpia.teaplant.org/download.html, respectively. The hidden Markov model (HMM) profiles PF06200 (TIFY domain) and PF09425 (JAZ domain) of the JAZ family were extracted from the Pfam database (http://pfam.sanger.ac.uk). Then these two HMM profiles were used to search the local tea protein database for target hits with the TIFY and JAZ domain by HMMER 3.0 (http://hmmer.janelia.org/). All non-redundant sequences with E-values lower than 1.0E-05 were selected. And the obtained candidates were further confirmed by the SMART web server (http://smart.emblheidelberg.del) and CD-search (https://www.ncbi.nlm.nih.gov/Structure/cdd/wrpsb.cgi). 

### 4.2. Phylogenetic Tree Construction and Sequence Alignment 

The JAZ protein sequences of *Arabidopsis thiatina*, *Oryza sativa*, *Vitis vinifera*, *Populus trichocarpa*, *Gossypium raimondii,* and *Camellia sinensis* were aligned using DNAMAN 7.0 (Lynnon Biosoft, America), and a phylogenetic tree was established using the neighbor-joining (NJ) method in the MEGA 7.0 software program with 1000 bootstrap replicates [45]. Multiple sequence alignment of the candidate CsJAZ proteins was also performed using DNAMAN 7.0.

### 4.3. Characterization of CsJAZ Genes and Proteins

The intron/exon of the *CsJAZs* were predicted using the TBtools [46] based on the comparison of CDS and genomic sequences. The online MEME (http://meme-suite.org/index.html) program was used to analyze the conserved motif structures of the proteins encoded by the *CsJAZ* genes. The promoter sequences were analyzed from approximately 2 kb upstream of the transcription start site of each gene, and the *cis*-elements were obtained using the PlantCARE database (http://bioinformatics.psb.ugent.be/webtools/plantcare/html/) [10]. CsJAZ proteins were annotated using BLAST against GO with an E-value threshold of 1E-5 [47]. The protein interaction networks were integrated into STRING software Version 11.0 (https://string-db.org/) [48]. 

### 4.4. Plant Materials, Growth Conditions, and Stress Treatments

One-year-old “Longjing43” tea seedlings were used as plant materials in the study. The seedlings were grown in greenhouse under 16 h light (25 °C)/8 h dark (20 °C) photoperiod, with 3600 Lx photos m^−2^·s^−2^ light intensity and 75% humidity for 2 weeks before treatment. For cold stress treatment, seedlings were transferred to a growth chamber at 4 °C and sampled at 0, 4, 12, and 36 h after treatment. Heat shock stress was applied after transfer to growth chamber at 42 °C and samples were collected at 0, 4, 12, and 36 h after treatment. For drought stress treatment, the seedlings were sprayed with 10% PEG 6000 and sampled at 0, 4, 12, and 36 h after treatment. Hormone treatments were performed with 100 µM methyl jasmonate (JAMe) and 100 µM gibberellin (GA3), respectively. Samples were collected at 0, 4, 12, and 36 h after MeJA or GA3 treatments. The tender leaf (TL), mature leaf (ML), stem (S), and root (R) of the seedlings grown under normal conditions were also sampled. For all conditions, including control, three biological replicates of each sample were collected. All samples were frozen immediately in liquid nitrogen and kept at -80 °C for total RNA extraction.

### 4.5. Total RNA Extraction and qRT–PCR Analysis

Total RNA was extracted from the samples according to our previous report [49]. The integrity of the RNA was assessed by 1.0% agarose gel electrophoresis. TransScript^®®^ One-Step gDNA Removal and cDNA Synthesis SuperMix (TransGen, Beijing, China) was used for genomic DNA digestion and first-strand cDNA synthesis. The expression analysis of the 12 *CsJAZ* genes was performed by qRT–PCR using SYBR Premix Ex Taq II kit (Takara, Kusatsu, Japan) with the Bio-Rad IQ5 Real Time PCR System (Bio-Rad, Hercules,CA, USA). The primer pairs used for qRT–PCR are listed in Table S4. *Camellia sinensis* GAPDH and *β*-actin were used as an internal control. The qRT–PCR conditions were as follows: 95 °C for 30 s, 40 cycles at 95 °C for 5 s, 60 °C for 30 s. After amplification, the melting curve and amplification curve were checked to evaluate specific amplification. For each gene, all experiments were repeated three times per sample. Relative gene expressions were calculated using the 2^−ΔΔCt^ method [50]. The transcripts levels are presented as the mean ± standard error mean (SEM). 

### 4.6. Pearson Correlation Analysis

The pairwise Pearson correlation coefficients (PCCs) and *p*-values of *CsJAZ* gene expression levels were calculated and visualized using the GraphPad Prism 7 software based on the qRT–PCR results. Gene co-regulatory networks were constructed by Cytoscape (version 3.7.1, Seattle, WA, USA) based on the PCCs of *CsJAZ* gene pairs with a *p*-value significance level of 0.05.

### 4.7. Subcellular Localization Prediction and Confirmation

The subcellular localization of the CsJAZ proteins was predicted using CELLO v.2.5 (http://cello.life.nctu.edu.tw/). For testing of the predicted nuclear localization, the full-length sequence of *CsJAZ3*, *CsJAZ10,* and *CsJAZ11* gene without the stop codon were amplified (primers were listed in Table S4) and then inserted into EGFP-fusion expression vector pCAMBIA 2300 using Trelief™ SoSoo Cloning Kit Ver.2 (TSINGKE, Beijing, China). Plasmids with gold powder were transferred to onion cells to analyze the locating signals. Empty vector 35S::EGFP was used as a control. An LSM800 confocal microscopy imaging system (Zeiss Co., Oberkochen, Germany) was used to observe the fluorescence images of EGFP fusion proteins. 

### 4.8. Spot Assays of E. coli Cells with CsJAZ3, CsJAZ10, and CsJAZ11 Genes in Different Abiotic Stress

The ORF of *CsJAZ3*, *CsJAZ10*, and *CsJAZ11* were amplified (primers were shown in Table S4) and inserted into pGEX-4T-1 using Trelief™ SoSoo Cloning Kit Ver.2 (TSINGKE, Beijing, China). The recombinant plasmid (pGEX-4T-1–*CsJAZ3*, pGEX-4T-1–*CsJAZ10,* and pGEX-4T-1–*CsJAZ11*) was obtained and then transformed into *E. coli* BL21 (DE3) competent cells. To study the expression of *CsJAZ3*, *CsJAZ10*, and *CsJAZ11* in *E. coli* in response to different abiotic conditions, spot assays were conducted in combination with heat treatment and treatments with PEG 6000, NaCl. All the experiments were done at least three times to make sure that the results were reliable and reproducible.

The *E. coli* BL21 cells containing pGEX-4T-1–*CsJAZ3*, pGEX-4T-1–*CsJAZ10*, pGEX-4T-1–*CsJAZ11* and pGEX-4T-1 (EV) were cultured in LB medium at 37 °C until they reached OD600 of 0.6. Then, isopropyl β-D-thiogalactoside (IPTG) was added to the cultures at a concentration of 1.0 mM and further cultured at 37 °C for another 12 h. The cultured cells were diluted to an OD600 of 0.6 and then diluted to 10^−3^- and 10^−4^-fold, respectively by using the LB medium [51]. For heat treatment, volume of 1 mL 10^−3^- and 10^−4^-fold dilutions were put into a temperature-controlled water bath (55 °C) for 0, 30, 60, and 90 min. Then ten microliters from each of the 10-3- and 10-4-fold dilutions was spotted onto LB plates containing 100 μg·mL^−1^ ampicillin. For PEG 6000 and NaCl treatments, ten microliters from each of the 10^−3^- and 10^−4^-fold dilutions was spotted onto LB plates containing different concentrations of PEG 6000 (5% and 10%) and NaCl (250, 500, and 750 mM). All the LB basal plates contained 100 μg·mL^−1^ ampicillin. Then all plates were cultured at 37 °C overnight and then photographed. These experiments were also done at least three times to assure that the results were reliable and reproducible. 

## 5. Conclusions 

In conclusion: 12 *JAZ* genes in tea plant were identified and their bioinformatics information, including the classification, protein motifs, and gene structure, gene promoters were investigated in this study. Quantitative RT–PCR analysis showed that 7 *CsJAZ* genes were preferentially expressed in roots. The relative expressions of *CsJAZ* genes revealed the potential role of *CsJAZ* genes in “LJ43” against low and high temperature, PEG, MeJA, and GA stress. Additionally, we identified several *CsJAZ* genes such as *CsJAZ4*, -*10*, -*11* that may be utilized as candidates for improving tea resistances to multiple stresses. In addition, the overexpression of *CsJAZ3*, *CsJAZ10-* and *CsJAZ11* in *E. coli* BL21 cells enhanced its growth under high temperature, NaCl, and PEG stimuli. The findings of the present study may be utilized in future research investigations on the function of JAZ family genes in tea plant. Further studies are required to reveal the molecular mechanisms of *CsJAZ* in response to abiotic stress at translational and post-translational levels by biochemical and genetic approaches. 

## Supplementary Materials

Supplementary materials can be found at https://www.mdpi.com/1422-0067/21/7/2433/s1.
