# Supplementary material for: Genome-Wide Analysis Reveals Stress and Hormone Responsive Patterns of JAZ Family Genes in Camellia Sinensis
Source: Int J Mol Sci. 2020 Mar 31;21(7):2433. doi: 10.3390/ijms21072433 (PMC7177655; doi:10.3390/ijms21072433)
Supplement: Supplementary file 1 [file ijms-21-02433-s001.pdf]

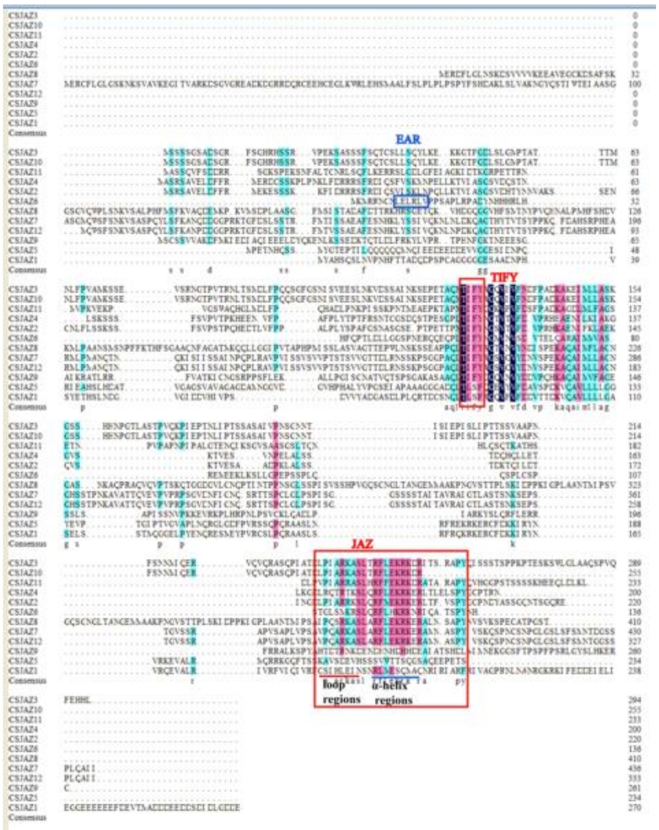

Figure S1. Multiple sequence alignment of the CsJAZ proteins. The TIFY and JAZ regions are indicated by a red box.

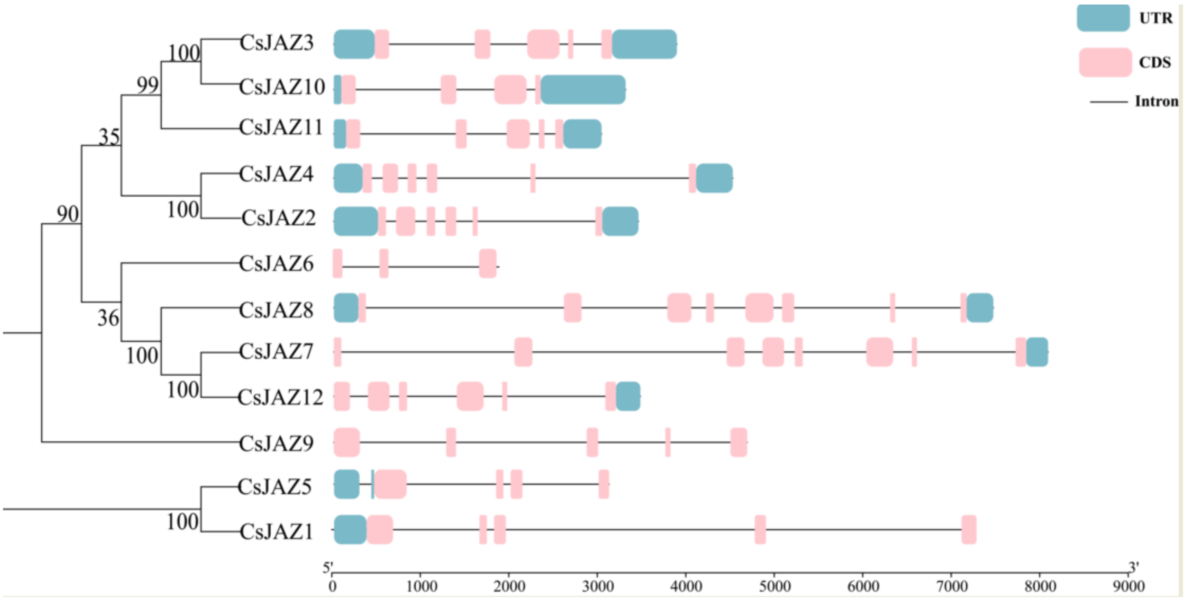

Figure S2. Phylogenetic relationships and gene structures of the CsJAZ family. Exons and introns were shown by filled boxes and single lines, respectively.

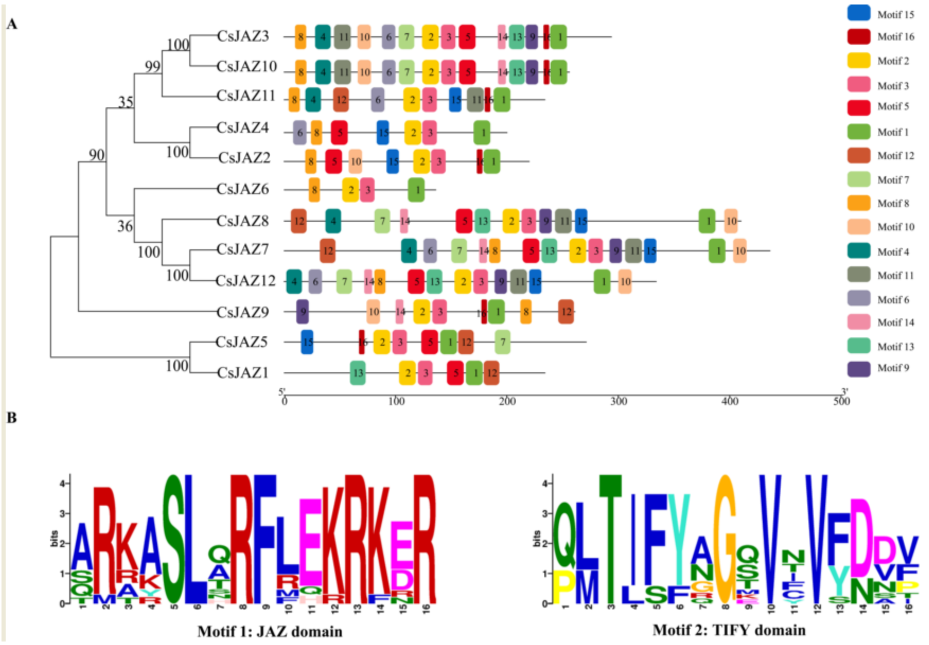

Fig. S3 Motif analysis of CsJAZ proteins. Each colored box represented conservation.

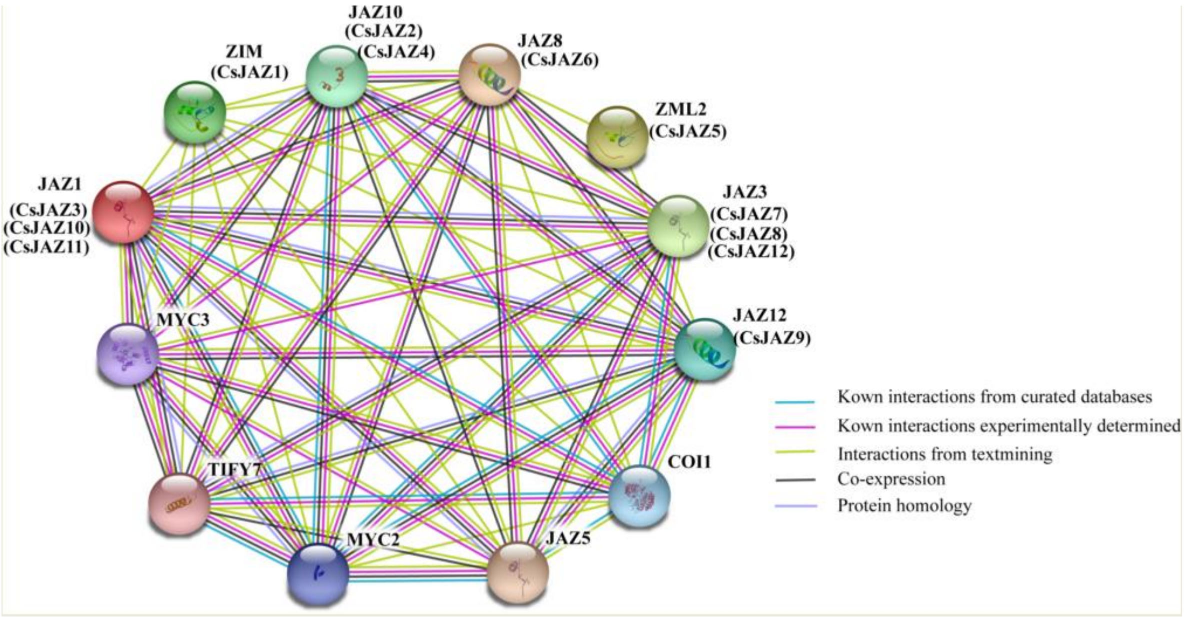

Fig. S4 Functional interaction networks of CsJAZ proteins.

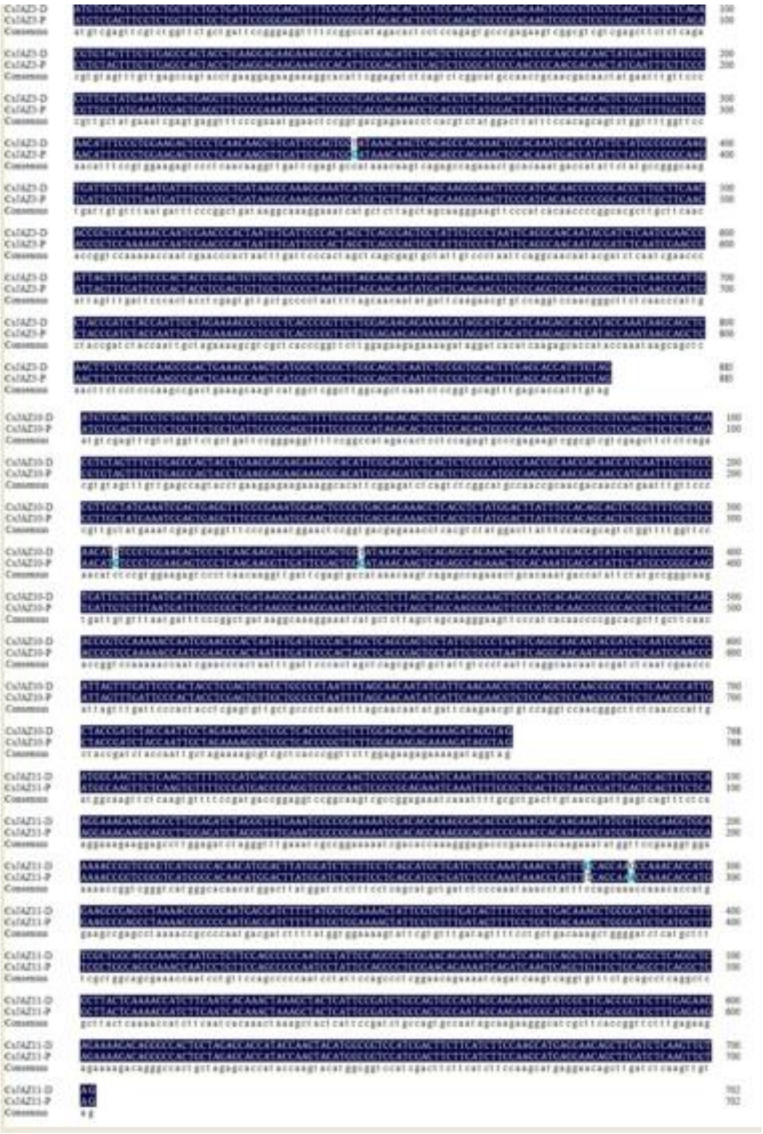

Fig. S5 The alignments of full-length sequence of *CsJAZ3*, *CsJAZ10* and *CsJAZ11* genes obtained by PCR and tea genome database. -D means that the sequence is from tea genome database. -P means that the sequence is obtained by the PCR amplification
